# The effect of life events, resilience, self-esteem, and coping styles on aggressive behavior among left-behind adolescents: Structural equation modeling

**DOI:** 10.3389/fpsyt.2023.991608

**Published:** 2023-01-30

**Authors:** Jie Zhang, Yifei Li, Juan Li, Mengmeng Lyu, Yiping Chen, Silan Yang, Chuxia Tan, Yingxiang Tao, Biyun Ye, Jingping Zhang, Fangqun Cheng, Ting Mao

**Affiliations:** ^1^Hunan University of Chinese Medicine, Changsha, China; ^2^Xiangya School of Nursing, Central South University, Changsha, China; ^3^National University of Singapore, Singapore, Singapore; ^4^Xiangtan Central Hospital, Xiangtan, China

**Keywords:** left-behind adolescents, aggressive behavior, life events, self-esteem, resilience, coping style

## Abstract

**Introduction:**

Without parental support, left-behind adolescents are more likely than their peers to experience negative emotions and demonstrate aggressive behavior in the same frustrating situation. However, research on this subject has been sparse. To fill this gap and identify potential targets for intervention, this study sought to examine the relationships among factors influencing left-behind adolescents’ aggressive behavior.

**Methods:**

A total of 751 left-behind adolescents were enrolled in a cross-sectional survey, with data collected using the Adolescent Self-Rating Life Events Checklist, Resilience Scale for Chinese Adolescents, Rosenberg Self-Esteem Scale, Coping Style Questionnaire, and Buss–Warren Aggression Questionnaire. The structural equation model was used for data analysis.

**Results:**

The results showed that left-behind adolescents reported higher levels of aggression. Further, the factors found to have a direct or indirect effect on aggressive behavior included life events, resilience, self-esteem, positive coping, negative coping, and household income. The results of confirmatory factor analysis indicated goodness of fit. In the face of negative life events, left-behind adolescents with high resilience, self-esteem, and positive coping were less likely to exhibit aggressive behavior (*P* < 0.05).

**Discussion:**

Left-behind adolescents can reduce their aggressive behavior by assuaging the adverse effects of life events via increased resilience and self-esteem as well as the adoption of positive coping strategies.

## 1. Introduction

Globally, nearly one in seven individuals is a migrant. The majority are labor migrants who originate from low- or middle-income countries (LMICs) and relocate in search of employment opportunities, either internationally or internally (e.g., rural to urban) (1). According to data released in 2019 by China’s National Bureau of Statistics, the total number of migrant workers in China had reached nearly 300 million, accounting for about one-fifth of the developing country’s total population (2). Further, constraints related to finances, housing, and urban school entrance requirements have led some migrant workers to leave their children in rural homes, thus forming a special group of minors: left-behind children (3).

China’s State Council defines left-behind children as minors under the age of 16 years who do not regularly live with their parents because one or both is away for work or incapable of guardianship (4). According to data released in 2018 by the Ministry of Education (5), the number of left-behind children in rural China has exceeded 15 million. Among this group, 91.3% have experienced varying degrees of mental and physical neglect and abuse, including sexual violence (6). These life events result in higher levels of mental health issues (i.e., anxiety, depression, suicidal tendencies) and aggressive behavior (i.e., violent crimes) in left-behind adolescents compared to minors from typical families (7, 8). Meanwhile, adolescence is an important stage in the development of individual personality, sociality, and values. Generally defined from 10 to 20 years of age, adolescence is the transitional period between childhood and adulthood (9). During this period, rebellious and aggressive behaviors are particularly prominent (10). Numerous studies have shown that left-behind adolescents are significantly more likely than their peers to display aggressive behavior in the same frustrating situation (3, 11, 12).

Highly aggressive behavior is detrimental to the physical and psychological health of the individual. Research shows that adolescents with high aggressiveness are more likely to have psychological problems and commit violent acts as adults, including self-harm and suicidal tendencies (13, 14). Although some researchers have investigated the aggressive behaviors of left-behind adolescents, most only conducted one-factor analysis of variance (ANOVA) or regression analysis of the influencing factors (3, 11, 12). Additional analysis is needed to support the development of targeted intervention plans. Therefore, we aimed to adopt structural equation modeling (SEM) to analyze the interrelationship and acting pathways of various factors affecting the aggressive behavior of left-behind adolescents. The goal of this study was to provide a reference conceptual framework for prevention and interventions to reduce aggressive behavior in this population.

### 1.1. Stress-coping model with left-behind adolescents

Left-behind adolescents are highly susceptible to psychological stress and, by extension, aggressive behavior, and other negative coping strategies (3). The cognitive-phenomenological-transactional (CPT) model (15, 16), derived from Lazarus and Folkman’s stress and coping theory, is the most widely used theoretical model in studies of left-behind adolescent psychology and behavior. It emphasizes the absolute role of cognitive evaluation in the process of coping with stress (17), which is consistent with the frustration-aggression theory proposed by Miller (18). As the first theory to delineate aggressive human behavior, the frustration-aggression theory proposes that aggressive behavior is a response to frustration (18). It is consistent with the three variables in the CPT theoretical model: stressors, environmental information, and individual-environmental interactions (15).

Based on the stress-coping model, negative life events faced by children act as initial stressors (19). In the intermediate stage that follows, the individual evaluates their environment to determine the presence and form of these stressors (15). Initial stressors can trigger cognitive evaluation and individual-environmental interactions, with the consequences of the latter being positive or negative (15). Thus, the intermediate stage reflects the role of individual cognition as well as the influence of environmental factors in the process of coping with stress. Due to a lack of family support (e.g., timely care from parents), left-behind adolescents usually face more negative life events and are more inclined to adopt negative coping tendencies, such as aggressive behavior (3, 6, 7). It is, therefore, crucial to examine the key factors associated with the intermediate stage to prevent and reduce the aggressive behavior of left-behind adolescents in response to negative life events.

### 1.2. Resilience, self-esteem, and coping style as mediators in the stress-coping model

Frustration responses are determined by the individual’s cognition and evaluation of frustration (20). Frustration response consists of emotion, self-esteem, and personality traits (21). Frustration–aggression theory holds that frustration can awaken individual emotions (22). Emotions to frustrations, such as positive and negative emotions, vary between individuals (23). Coping can be defined as a cognitive–behavioral activity of a person to manage his/her emotions in a stressful situation (17). In the same setbacks, adolescents with positive emotions tend to adopt positive coping styles and less aggressive behaviors (23, 24). Self-esteem is a positive evaluation of oneself as valuable (25). Teens with low self-esteem are likely to produce aggression (26, 27). Resilience refers to the dynamic process in which an individual can adapt well to a dangerous environment (28) and is significantly positively correlated with positive personality traits (29). Adolescents with high levels of resilience are likely to control and stabilize their emotions after a setback, resulting in reduced aggressive behaviors (30).

Although not studied in the comprehensive model, resilience, self-esteem, and coping style are potentially intermediary factors in the relationship between life events and aggressive behavior choices of left-behind adolescents.

### 1.3. Relationship between resilience, self-esteem, and coping style

Several studies on left-behind adolescents found correlations between resilience, self-esteem, and coping style. Individuals with high levels of resilience and self-esteem are likely to perceive positive external stimuli and thus respond positively (28, 31), furthermore, self-esteem is positively correlated with resilience (32). Therefore, we hypothesize that self-esteem and resilience are positively correlated with positive coping and are negatively correlated with negative coping, that is, self-esteem has positive associations with resilience. Negative and positive coping styles are contradictory.

Zhang and Qiu (33) found that resilience and coping style can negatively predict adolescents’ aggressive behavior and that coping style can also influence this aggressive behavior through the mediating effect of resilience. Wang and Zhang (34) found that self-esteem and coping styles could predict aggressive behavior in adolescents, whereas coping styles did not work as mediators in the relationship between self-esteem and aggressive behavior. Therefore, although resilience, self-esteem, and coping style can influence the aggressive behavior of adolescents, their influence pathways and intensity should be studied further.

### 1.4. Aim of the study

**FIGURE 1:**
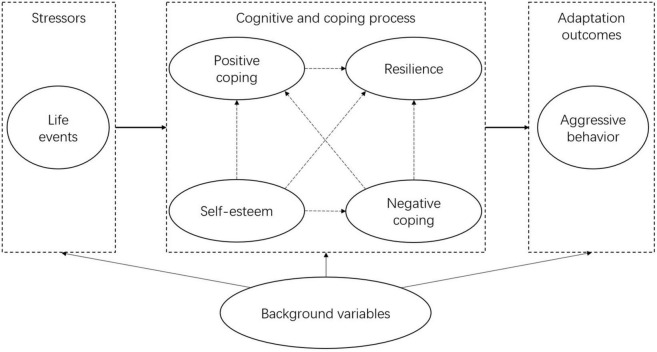
Hypothetical model A.

**FIGURE 2:**
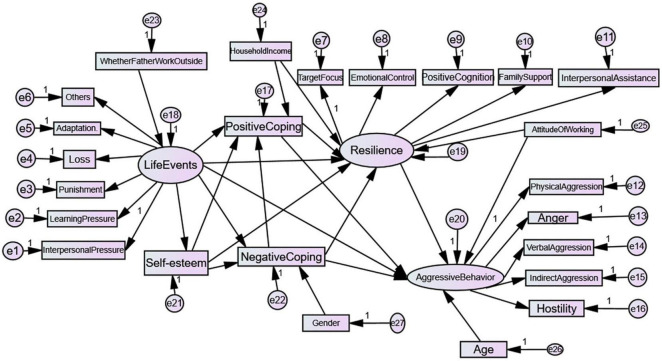
Hypothetical model B.

A comprehensive analysis of the interaction between the influencing factors of life events, resilience, self-esteem, and coping style is important to formulating targeted programs aimed at reducing left-behind adolescents’ aggressive behavior and promoting their physical and mental health. Based on previous literature reviews, we built hypothetical models A and B. In hypothetical model A, life events of left-behind adolescents are referred to as stressors. Resilience, self-esteem, and coping style are the intermediary variables, and aggressive behaviors are the outcomes (Figure 1). In hypothetical model B, we assumed the potential relationships between these variables (Figure 2). This study aimed to examine the relationships between life events, resilience, self-esteem, and coping styles and their effect on left-behind adolescents’ aggressive behavior by using SEM. The findings of this study contribute to the development of targeted interventions in left-behind adolescents’ aggressive behavior.

## 2. Materials and methods

### 2.1. Design and sample

This study adopted a cross-sectional design and multi-stage stratified random sampling. First, one city from each of the four administrative regions of Hunan province was randomly selected. Second, in each selected city, a rural junior high school was selected randomly; thus, four rural junior high schools were selected in total. The inclusion criteria were adolescents: (1) aged between 10 and 16 years, (2) who had both or one of the parents who had been away for more than half a year, and (3) who were willing to participate in the study. We excluded adolescents with cognitive impairment, and data were collected between 13 and 17 April, 2020.

A minimum sample size of 200 is typically recommended for SEM analysis. A popular *a priori* sample size calculator publicly designed for calculating SEM sample sizes was applied^1^. With a moderate effect (0.3), a power value of 0.95, and an α of 0.05 and including 3 latent and 24 observed variables (all observed indicators and socio-demographic variables), the minimum sample size for this study is 700.

Before data collection, we received permission from the headmaster and head teacher of each school. With the help of the head teachers, we gathered the participants in several classrooms and distributed questionnaires to them without the presence of teachers. After all the questionnaires were collected, we divided the participants into left-behind and non-left-behind adolescents by asking them, “Did one or both of your parents out-migrate for work for at least 6 months?” Based on their responses, the questionnaires of the left-behind adolescents were selected for analysis. Finally, we recruited 789 left-behind adolescents and obtained 751 valid responses, corresponding to a response rate of 95.18%.

### 2.2. Measuring instruments

#### 2.2.1. Socio-demographic characteristics

Socio-demographic characteristics were collected, such as gender, age, grade, whether the only child, household income, parents going out, the attitude of adolescents toward their parents’ migrant work, and other general conditions of left-behind adolescents.

#### 2.2.2. Adolescent Self-rating Life Events Checklist

Liu et al.’s (35) Adolescent Self-rating Life Events Checklist (ASLEC) was applied to measure the frequency and intensity of negative life events experienced by left-behind adolescents. This scale includes 27 items exploring five domains: punishment, loss, interpersonal pressure, learning pressure, and adaptation. ASLEC adopts a six-point Likert scale from 1 (“not at all”) to 6 (“very much”). The higher the score, the more negative events they faced. The scale has good reliability and validity, with Cronbach’s α, split-half reliability, and test–retest reliability at 0.95, 0.88, and 0.69, respectively. In this study, Cronbach’s α was 0.91.

#### 2.2.3. Resilience Scale for Chinese Adolescents

Hu and Gan’s (36) Resilience Scale for Chinese Adolescents was used to measure the resilience of left-behind adolescents. This scale includes 27 items covering five factors: target focus, emotional control, positive cognition, family support, and interpersonal assistance. It adopts a five-point scale (1–5, representing from “never” to “always”), with higher scores reflecting greater resilience. Cronbach’s α was 0.85 for the Chinese version and 0.82 for this study.

#### 2.2.4. Rosenberg Self-Esteem Scale

The Rosenberg Self-Esteem Scale (RSES) was designed by Rosenberg (37) and translated by Ji and Yu (38). RSES is an extensive assessment of self-esteem, with high authority. It includes 10 items, rated on a four-point Likert scale ranging from 1 (“strongly disagree”) to 4 (“strongly agree”). The overall self-esteem factor can be calculated with the sum score ranging from 10 to 40. A higher score indicates higher self-esteem. Cronbach’s α was 0.78 for the Chinese version and 0.80 for this study.

#### 2.2.5. Coping Style Questionnaire

Coping style was measured by Xie’s (39) 20-item simplified Coping Style Questionnaire (CSQ). Responses for each question range from 0 to 3 (0 = “never,” 1 = “seldom,” 2 = “often,” and 3 = “always”). This scale includes two subscales to assess positive (items 1–12) and negative coping (items 13–20). A high score on each dimension indicates frequent usage of this type of coping. The internal consistency of the two subscales and the entire scale was 0.90, 0.89, and 0.78, respectively. In this study, Cronbach’s α of the scale was 0.80.

#### 2.2.6. Buss–Warren Aggression Questionnaire

The Buss–Warren Aggression Questionnaire (BWAQ) was designed by Buss and Warren (40) and translated by Wang et al. (41). The 34-item scale is a self-assessment questionnaire with five domains: physical aggression, anger, verbal aggression, indirect aggression, and hostility. The items were rated on a five-point Likert scale ranging from 1 (“strongly disagree”) to 5 (“strongly agree”). The higher the score, the more aggressive the participants were. Cronbach’s α of the total scale was 0.86, and that of each subscale ranged from 0.51 to 0.75. In this study, Cronbach’s α was 0.920.

### 2.7. Ethical considerations

Before the data collection, ethics approval was obtained from the Ethics Committee of our university (No. E201946). Permission to collect data was granted by the principal and head teacher of each school before conducting the survey. The participants were informed of the purpose, method, and considerations of the study and that they could quit at any time during the filling process in the survey. They further signed an informed consent form. The cover page of the questionnaire contained contact information for psychological consultations, should they need to.

### 2.8. Data analysis

Statistical analyses were conducted using IBM SPSS Statistics version 20.0 and AMOS 20.0 (IBM, Chicago, IL, USA). Descriptive data were presented as means and standard deviations. Pearson’s correlation analyses were used to identify the correlations between the variables. ANOVA, independent sample *t*-tests, and multiple comparisons (LSD) were used to analyze the associations between socio-demographic characteristics and psychosocial variables in the model. This method helped to identify covariates in the model. Hypothesized model B comprised latent and observed variables (Figure 2). To optimize SEM, the incremental fit index (IFI), comparative fit index (CFI), gamma goodness-of-fit index (GFI), normed fit index (NFI), and root mean square error of approximation (RMSEA) were used as model fit indicators. Values of CFI, IFI, NFI, and GFI > 0.90 are considered to reflect a good model fit. RMSEA values < 0.05 suggest a good fit, whereas values up to 0.08 indicate reasonable errors of approximation and an acceptable fit. The parsimony of the hypothetical model was improved by eliminating standardized path coefficients with small effects (absolute values < 0.10).

## 3. Results

### 3.1. Analyzing basic participant characteristics

Table 1 shows significant differences in socio-demographic characteristics between variable scores in the model. Age, gender, household income, father out for work, and the attitude of adolescents toward their parents’ migrant work influenced at least one variable.

**TABLE 1:** Relationships between socio-demographic characteristics and variable scores of all variables.

Variables	*N* (%)	Life events	Resilience	Pairwise comparison	Self-esteem	Positive coping	Pairwise comparison	Negative coping	Aggressive behavior	Pairwise comparison
**Age (years)**										
11	6 (0.8%)	73.00 ± 20.00	87.00 ± 5.80		25.33 ± 3.78	15.33 ± 4.59		6.83 ± 3.31	74.33 ± 17.65*	a1 < c1*
12^a1^	114 (15.2%)	72.95 ± 25.46	93.19 ± 17.87		28.17 ± 5.40	19.05 ± 6.60		8.04 ± 4.86	76.55 ± 22.41	a1 < d1*
13^b1^	266 (35.4%)	77.69 ± 22.78	96.69 ± 13.31		27.34 ± 5.43	19.04 ± 6.53		8.84 ± 4.56	81.09 ± 22.99	b1 < d1*
14^c1^	273 (36.4%)	73.67 ± 22.14	91.92 ± 14.70		28.09 ± 4.84	18.90 ± 6.76		8.63 ± 4.67	81.89 ± 23.14	
15^d1^	89 (11.9%)	78.15 ± 26.83	87.34 ± 13.01		27.61 ± 4.54	17.50 ± 7.32		8.91 ± 4.52	87.06 ± 21.89	
16	3 (0.4%)	67.67 ± 23.03	92.33 ± 11.68		27.67 ± 4.04	16.67 ± 13.01		13.00 ± 5.20	61.54 ± 19.30	
**Gender**										
Male	379 (50.5%)	76.22 ± 23.35	91.62 ± 14.42		27.65 ± 5.18	18.46 ± 6.56		9.10 ± 4.67*	82.62 ± 22.87	
Female	372 (49.5%)	74.68 ± 23.65	91.25 ± 15.40		27.86 ± 5.01	19.08 ± 6.91		8.19 ± 4.57	80.03 ± 23.01	
**Grade**										
7	221 (29.4%)	77.13 ± 24.88	91.31 ± 16.27		27.38 ± 5.73	18.46 ± 6.16		8.78 ± 4.72	78.91 ± 22.36	
8	263 (35.0%)	74.29 ± 20.96	92.03 ± 13.56		27.71 ± 4.75	19.00 ± 7.00		8.58 ± 4.52	80.15 ± 23.32	
9	267 (35.6%)	75.65 24.61	90.95 ± 14.86		28.13 4.86	18.91 6.81		8.67 ± 4.65	84.34 ± 22.75	
**Only child**										
Yes	112 (14.9%)	78.49 ± 27.86	91.98 ± 15.16		27.65 ± 4.66	18.45 ± 8.15		8.14 ± 4.09	80.40 ± 23.59	
No	639 (85.1%)	75.18 ± 22.70	91.32 ± 14.74		27.75 ± 5.17	18.85 ± 6.42		8.74 ± 4.70	81.49 ± 22.89	
**Household income/y**										
< 10k^a2^	125 (16.6%)	78.02 ± 24.20	91.24 ± 14.46*	a2<b2*	28.50 ± 4.68	19.90 ± 6.49*	a2 > d2*	9.35 ± 4.64	81.55 ± 24.76	
10k–20k^b2^	122 (16.2%)	73.61 ± 24.75	95.64 ± 16.53	b2 > d2*	28.03 ± 6.36	20.80 ± 6.89	b2 > d2*	8.27 ± 4.99	76.95 ± 22.68	
>20k^c2^	116 (15.4%)	75.89 ± 22.17	93.62 ± 14.89	c2>d2*	27.85 ± 4.58	19.32 ± 7.31	c2 > d2*	9.80 ± 4.96	82.12 ± 24.23	
Unclear^d2^	388 (51.7%)	75.28 ± 23.23	89.60 ± 14.13		27.34 ± 4.89	17.66 ± 6.32		8.64 ± 4.45	82.15 ± 22.01	
**Father out for work**										
Yes	684 (91.1%)	76.52 ± 23.48*	91.38 ± 14.93		27.72 ± 5.12	18.75 ± 6.65		8.65 ± 4.66	81.40 ± 22.84	
No	67 (8.9%)	68.79 ± 20.66	91.04 ± 12.93		27.90 ± 4.86	19.14 ± 7.03		9.25 ± 4.19	82.72 ± 23.43	
**Mother out for work**										
Yes	474 (63.1%)	75.81 ± 22.99	91.03 ± 14.08		27.65 ± 4.91	18.51 ± 6.64		8.86 ± 4.65	81.22 ± 22.42	
No	277 (36.9%)	75.55 ± 24.31	92.21 ± 15.70		27.89 ± 5.38	19.24 ± 6.65		8.36 ± 4.58	81.19 ± 24.08	
**Attitude of the adolescents toward their parents’ migrant work**										
Strongly agree^a3^	91 (12.1%)	71.53 ± 24.08	94.50 ± 15.49*	a3 > c3*	28.38 ± 5.93	19.81 ± 7.04		8.69 ± 5.02	75.58 ± 24.39*	a3 < c3*
Agree^b3^	426 (56.7%)	75.74 ± 23.07	92.07 ± 14.30	a3 > d3*	27.93 ± 4.80	18.73 ± 6.49		8.64 ± 4.49	80.12 ± 21.75	a3 < d3*
Indifferent^c3^	104 (13.8%)	76.39 ± 23.40	87.80 ± 15.67	a3 > e3*	26.77 ± 5.71	17.50 ± 6.49		9.22 ± 4.78	87.40 ± 22.58	b3 < c3*
Disagree^d3^	105 (14.0%)	76.48 ± 22.38	89.88 ± 15.31	b3 > c3*	27.42 ± 4.70	19.16 ± 6.76		8.82 ± 4.62	86.51 ± 24.61	b3 < d3*
Strongly disagree^e3^	25 (3.3%)	81.00 ± 31.37	86.91 ± 11.44		27.40 ± 5.56	19.01 ± 7.78		8.12 ± 0.75	82.32 ± 22.60	

*P < 0.05; these scores were significantly higher or lower than those for other groups within the socio-demographic characteristics.

### 3.2. Descriptive analysis of left-behind adolescents’ aggressive behavior and other variables

Table 2 shows the average scores of life events, resilience, self-esteem, positive coping styles, negative coping styles, and aggressive behaviors of left-behind adolescents at (75.58 ± 23.47), (91.41 ± 14.85), (27.76 ± 5.10), (18.78 ± 6.71), (8.66 ± 4.62), and (81.28 ± 22.91), respectively.

**TABLE 2:** Descriptive statistics of the measured variables (*n* = 751).

Variables	Mean	SD	Median	Range
Life events	75.58	23.47	73.00	28.00–147.00
Resilience	91.41	14.85	89.00	50.00–131.00
Self-esteem	27.76	5.10	28.00	10.00–40.00
Positive coping	18.78	6.71	19.00	0.00–36.00
Negative coping	8.66	4.62	8.00	0.00–22.00
Aggressive behavior	81.28	22.91	82.00	37.00–152.00

Above table shows the descriptive analysis of resilience, life events, self-esteem, coping styles, and aggressive behaviors of left-behind adolescents.

### 3.3. Relationship between left-behind adolescents’ aggressive behavior and other variables

Table 3 shows the relationships between life events, resilience, self-esteem, coping styles, and aggressive behaviors of left-behind adolescents. Life events had negative associations with self-esteem, positive associations with negative coping and aggressive behaviors, and no associations with positive coping. The resilience of left-behind adolescents was negatively correlated with life events, negative coping, and aggressive behaviors but positively correlated with self-esteem and positive coping. Self-esteem positively affected positive coping but negatively affected negative coping and aggressive behavior. Positive coping positively affected negative coping but not aggressive behavior. Negative coping was positively correlated with aggressive behavior.

**TABLE 3:** Correlations among the measured variables (*n* = 751).

Variables	Life events	Resilience	Self-esteem	Positive coping	Negative coping	Aggressive behavior
Life events	1					
Resilience	−0.363^a^	1				
Self-esteem	−0.373^a^	0.624^a^	1			
Positive coping	−0.025^b^	0.498^a^	0.430^a^	1		
Negative coping	0.283^a^	−0.255^a^	−0.174^a^	0.228^a^	1	
Aggressive behavior	0.416^b^	−0.450^a^	−0.348^a^	−0.028^b^	0.429^a^	1

Above table shows the relationship among resilience, life events, self-esteem, coping styles, and aggressive behaviors of left-behind adolescents.

^a^*P* = 0.000; ^b^*P* > 0.05.

### 3.4. Structural equation modeling

**FIGURE 3:**
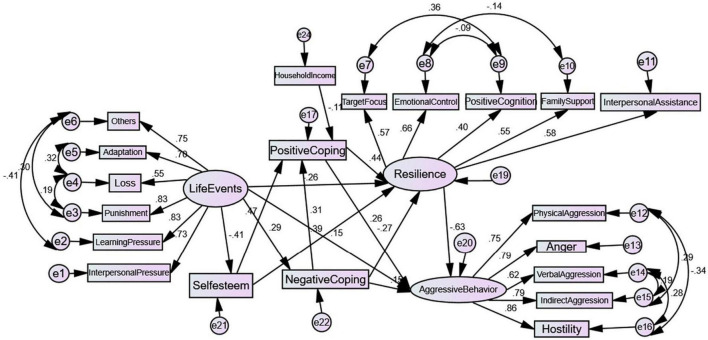
Modified model.

In this model, we used the subscales of coping styles, positive coping, and negative coping as observed variables. We conducted the confirmatory factor analysis of this scale. Before the analysis, the scale showed good indices (KMO ≥ 0.88; Bartlett’s statistic < 0.01), indicating its adequate properties for confirmatory factor analysis. The results of confirmatory factor analysis indicated good fit indices of the scale (GFI ≥ 0.90; RMSR ≤ 0.08; NFI, RFI, IFI, and CFI ≥ 0.90), demonstrating that the use of the instrument was legitimate. SEM was initially tested, and after removing pathways with small effects (absolute values < 0.10), according to the modify induce, several residual correlations have been added. Then, a modified model with a good model fit was obtained (Figure 3). The model parameters included χ^2^ = 687.50, DF = 150, χ^2^/DF = 4.583 < 5, GFI = 0.910 > 0.9, NFI = 0.903 > 0.9, IFI = 0.923 > 0.9, CFI = 0.922 > 0.9, RMSEA = 0.069 < 0.8 (Table 4). Although *P* < 0.05, all other measurement models had good fit indices, and the sample size was relatively large. Therefore, this model was acceptable (42). Figure 3 presents the standardized parameters of the final model.

**TABLE 4:** Comparison of model fit for the modified model to the hypothetical model.

Model	*χ^2^* (*P*)	df	*χ^2^*/df	GFI	NFI	IFI	CFI	RMSEA
Reference	>0.05		<5	0.9–1	0.9–1	0.9–1	0.9–1	<0.08
Hypothetical	1,469.99 (0.00)	239	6.151	0.842	0.803	0.830	0.829	0.083
Modified	687.50 (0.00)	150	4.583	0.910	0.903	0.923	0.922	0.069

Above table shows the parameters of the model. GFI, goodness-of-fit index; NFI, normed fit index; IFI, incremental fit index; CFI, comparative of fit index; df, degree of freedom; RMSEA, root mean square error of approximation.

Tables 5, 6 show the direct, indirect, and total effects of the relevant factors. Life events (effect value = 0.508), resilience (−0.626), positive coping (−0.019), negative coping (0.313), self-esteem (−0.253), and household income (0.002) have different direct and indirect effects on aggressive behavior of left-behind adolescents. Self-esteem, resilience, positive coping, and negative coping are the important mediating factors in the relationship between life events and left-behind adolescents’ aggressive behavior.

**TABLE 5:** Standardized direct, indirect, and total effects for the modified model.

Endogenous variables	Exogenous variables	Standardized direct effects	Standardized indirect effects	Standardized total effects
Aggressive behavior	Resilience	−0.626		−0.626
	Positive coping	0.256	−0.275	−0.019
	Negative coping	0.148	0.165	0.313
	Life events	0.147	0.361	0.508
	Household income		0.002	0.002
	Self-esteem		−0.253	−0.253
Resilience	Positive coping	0.439		0.439
	Life events	−0.263	−0.287	−0.550
	Negative coping	−0.273	0.135	−0.138
	Self-esteem	0.391	0.208	0.599
	Household income		−0.050	−0050
Positive coping	Self-esteem	0.473		0.473
	Negative coping	0.307		0.307
	Household income	−0.113		−0.113
	Life events		−0.104	−0.104
Negative coping	Life events	0.294		0.294
Self-esteem	Life events	−0.411		−0.411

**TABLE 6:** Maximum likelihood estimates of the modified model.

Pathway	Non-standardized coefficients	Standardized coefficients	Standard errors	Critical ratio	*P*
Self-esteem←Life events	−0.594	−0.411	0.055	−10.761	0.000
Negative coping←Life events	0.385	0.294	0.050	7.655	0.000
Positive coping←Negative coping	0.446	0.307	0.045	9.932	0.000
Positive coping←Self-esteem	0.624	0.473	0.041	15.319	0.000
Positive coping←Household income	−0.657	−0.113	0.178	−3.700	0.000
Resilience←Positive coping	0.157	0.439	0.015	10.560	0.000
Resilience←Negative coping	−0.142	−0.273	0.019	−7.622	0.000
Resilience←Self-esteem	0.184	0.391	0.019	9.522	0.000
Resilience←Life events	−0.179	−0.263	0.027	−6.645	0.000
Aggressive behavior←Resilience	−1.314	−0.626	0.184	−7.131	0.000
Aggressive behavior←Positive coping	0.192	0.256	0.045	4.307	0.000
Aggressive behavior←Negative coping	0.162	0.148	0.048	3.385	0.000
Aggressive behavior←Life events	0.211	0.147	0.071	2.966	0.003

## 4. Discussion

### 4.1. Analysis of the status and influencing factors of left-behind adolescents’ aggressive behavior

The results showed that the average score of left-behind adolescents’ aggressive behavior (81.28 ± 22.91) was at a high level in the standardized norm of Chinese adolescents’ aggressive behavior (80-88) (43): 11 years old = (69.42 ± 17.34); 12 years = (69.95 ± 16.69); 13 years = (71.35 ± 16.69); 14 years = (72.41 ± 16.49); 15 years = (72.26 ± 16.35); and 16 years = (73.16 ± 16.13). In our study, the scores of left-behind adolescents of all ages, except for 16 years old, were higher than the norm of Chinese adolescents’ aggressive behavior. However, our study participants aged 11 and 16 were few. Their scores for aggressive behavior did not significantly differ from those of other age groups. Therefore, left-behind adolescents have higher aggressive behaviors than ordinary adolescents.

Our analysis revealed that aggressive behavior was different depending on age between age and the attitude of adolescents toward their parents’ migrant work. Further analysis showed that among the age groups, excluding the underrepresented 11- and 16-year-olds, the adolescents’ aggressive behavior scores increased with age. The scores of aggressive behavior among 12-year-olds were significantly lower than those among 14- and 15-year-olds. The scores of aggressive behavior among 13-year-olds were significantly lower than those among 14-year-olds. This result may be related to the psychological characteristics of adolescents. Adolescence is widely known as an important period in the physical and psychological development of individuals. Previous studies have found that due to physical and mental changes, the psychological maturity of individuals shows a transient decline from late childhood to early adolescence and then rises again during late adolescence or adulthood (44). Adolescents’ aggressive behavior may be related to their psychological immaturity. Romero and Alonso (45) confirmed that children’s negative emotions and aggressive behavior increased with age during adolescence.

In addition, our study found that adolescents who strongly agree and agree for their parents to work outside the home were significantly less aggressive than those who disagree and feel indifferent. The resilience of adolescents who strongly agree for their parents to go out to work was significantly higher than those who feel indifferent, disagree, and strongly disagree. This result may be because left-behind adolescents who agree their parents go out to work have higher psychological maturity. Thus, they can view problems more rationally, better understand their parents, and are less prone to negative emotions. Therefore, teachers, parents, and temporary guardians should not only pay attention to the psychological changes of adolescents over time but also strengthen communication with adolescents to enable them to understand their parents, that is, although they cannot be with them, their parents love them. This action will improve the resilience of left-behind adolescents and reduce their aggressive behavior.

In this study, left-behind adolescents whose fathers were out for work experienced more negative life events than the rest of the population, which is consistent with the results of An (46). These data showed the impact of fathers’ absence on the personality of left-behind children. Feng (47) demonstrated that the father’s absence could reduce the social adaptability and anti-frustration ability of left-behind children, making them more susceptible to negative life events. In our study, the absence of mothers did not affect left-behind adolescents. This result may be because fathers play a protective role in children’s development. Additionally, left-behind children are more likely to suffer physical and mental abuse than non-left-behind children (6). Therefore, the presence of fathers may bring left-behind adolescents security that mothers cannot provide. Zhu’s (48) study also confirmed that the absence of a father was more detrimental to children after age three than the absence of a mother.

The study found significant differences in negative coping among left-behind adolescents of different genders, with left-behind girls reporting less negative coping, which was consistent with Huang’s (49) study. This result may be related to adolescent girls’ more psychologically mature and rational traits when faced with stressful problems. They are willing to relieve their stress by seeking help from others; thus, they are less likely to use negative coping strategies. In contrast, boys are less willing to show their weakness and lack self-confidence when encountering problems. Adolescents are in the stage of high self-awareness but cannot solve problems. Thus, they are more likely to choose negative coping in the face of stressful events.

Resilience and positive coping showed significant differences between adolescents with different household incomes. The resilience of left-behind adolescents with an annual household income of less than 10,000 yuan is significantly lower than those with an annual household income of 10,000–20,000 yuan. This result may be related to the economic status of the family and the negative experiences that adolescents experience at home (50, 51). Several studies (50, 51) have shown that the family environment has an important impact on the psychological development of adolescents, whereas the material environment is one of the important components. Thus, poor family environments may increase the psychological pressure on left-behind adolescents and damage their resilience.

In addition, left-behind adolescents who do know their household income have significantly lower resilience and positive coping than those otherwise, which may be due to two reasons. First, adolescents are less psychologically mature and lack an understanding of the difficulties of their families and parents. They may have lower resilience and positive coping because they do not understand why their parents go out to work and therefore have more negative emotions.

Second, adolescents are overly psychologically mature. They may be concerned about causing a heavy burden to their family because they are not aware of their household income. They may also choose to bear the burden when faced with frustrating events due to their reluctance to trouble the family. In the long run, this situation will damage their resilience and positive coping. Therefore, when left-behind adolescents are experiencing negative emotions because of their household income, their particular category should be determined. If the adolescents belong to the low household income category, they should be taught that temporary poverty can be managed and that they determine their future. When dealing with left-behind adolescents with low psychological maturity, their growth and development should be supported through education and making them understand that their parents’ responsibilities are not easy. For mature adolescents, communication with them should be improved, allowing them to understand that worrying is useless and that studying hard is the right thing to do.

### 4.2. Analysis of left-behind adolescents’ aggressive behavior and its relationship with other measured variables

The correlation analysis showed that aggressive behavior was positively correlated with negative events and negative coping. This result is consistent with previous research (52) and may be related to the adverse effects of negative events on adolescent mood and behavior as well as negative cognition (53). In addition, the negative correlation between left-behind adolescents’ aggressive behavior and resilience and self-esteem was consistent with a previous study (54). Left-behind adolescents with high resilience and self-esteem have been shown to produce few hostile emotions and aggressive behaviors when facing stressful or negative events. Therefore, children’s aggressive behaviors can be effectively reduced by increasing left-behind adolescents’ resilience and self-esteem and teaching them to choose positive coping ways when facing stressful events. Parents, teachers, and other guardians should pay attention to left-behind adolescents’ development of resilience and self-esteem during their education. For example, their self-esteem can be enhanced through encouragement and praise as well as teaching them to understand correctly the pressure and helping them build courage, thus improving their resilience. Good resilience and self-esteem can reduce left-behind adolescents’ aggressive behavior. Subsequently, SEM was used to clarify further the strength and pathways of the influence of each variable on the aggressive behavior of left-behind adolescents.

### 4.3. Structural equation modeling of left-behind adolescents’ aggressive behavior

According to the results of SEM, negative life events greatly influence the aggressive behavior of left-behind adolescents (0.508). Increased negative life events was associated with increased aggressive behavior of left-behind adolescents, which is consistent with Yang’s (55) result. Adolescence is a critical period for physical and mental development. Due to their immature minds and lack of proper guidance from parents, left-behind adolescents during this period are more likely to have aggressive behaviors stimulated by negative life events than non-left-behind adolescents (16). Therefore, the impact of negative life events should be reduced before preventing the occurrence of left-behind adolescents’ aggressive behavior.

Our results showed that resilience, self-esteem, positive coping, and negative coping played an important mediating role in the relationship between life events and aggressive behaviors of left-behind adolescents. In addition to the direct impact (0.147), the impact of life events on left-behind adolescents’ aggressive behavior is mainly derived from the indirect effects (0.361) of resilience (0.165), self-esteem (0.104), and negative coping (0.092). Moreover, resilience (−0.626), self-esteem (−0.253), negative coping (0.313), and positive coping (−0.019) can also influence the aggressive behaviors of left-behind adolescents to a certain extent through direct or indirect paths. Similarly, left-behind adolescents with high self-esteem, resilience, and positive coping tendency are less likely to have aggressive behaviors, whereas those with high negative coping tendencies are more likely to have aggressive behaviors. This result is consistent with Qi’s (56) study.

Resilience had the greatest impact on the aggressive behavior of left-behind adolescents and only had a direct negative effect, which is consistent with previous research (57). This result may be because good resilience can moderate the individual’s perception of negative information (58). People with high resilience are likely to view things positively. As a result, they may be more likely to adapt and recuperate when they encounter stressful events.

Self-esteem had an indirect negative effect on the aggressive behavior of left-behind adolescents, which is consistent with previous research (59). This result may be related to the concept that people with low self-esteem have negative self-evaluations and are sensitive to external events, making them prone to psychological problems and aggression (26).

Coping style had direct and indirect effects on aggression. A positive coping style was negatively correlated with aggression, whereas a negative coping style was positively correlated with aggression, which is consistent with Sun et al.’s (60) finding. Accordingly, cultivating the positive coping ability of adolescents can reduce their aggressive behavior. Therefore, teaching left-behind adolescents from a positive perspective is necessary. For example, when faced with stressful events, guiding them to solve problems from a positive perspective can be helpful.

In addition, household income had a weak indirect positive impact on the aggressive behavior of left-behind adolescents (0.002). This result may be because adolescents with low household incomes are likely to face life pressure (61). The psychological changes of adolescents from lower-income families should be monitored, providing them with life and psychological help. Moreover, adolescents who do not know their household income should be guided properly to let them understand their parents’ stress and encourage them to choose positive ways of coping.

Therefore, parents, teachers, and other guardians should pay attention to the emotional changes of left-behind adolescents. They should implement effective psychological interventions to improve left-behind adolescents’ resilience, self-esteem, and positive coping tendency and reduce the impact of negative life events on them to prevent the occurrence of aggressive behaviors.

## 5. Limitations

First, causal relationships between the variables were constructed based on theoretical analysis and the literature review because of the cross-sectional design and thus should be interpreted with caution. Second, we only investigated the impact of life events and internal psychological traits on left-behind adolescents’ aggressive behavior and did not analyze their external social support. Future research could apply a longitudinal design to test additional variables influencing the aggressive behavior of left-behind children at different periods.

## 6. Conclusion

This study used stress–coping theory to examine the factors that may influence the aggressive behavior of left-behind adolescents and further analyzed the pathways of action and intensity of the influencing factors through SEM. This approach provided reliable evidence and support for the development of subsequent psychological interventions for left-behind adolescents.

This study detected that the status of aggressive behavior among left-behind adolescents is significantly above the average. Life events, resilience, self-esteem, positive coping, and negative coping have direct and indirect effects on the aggressive behavior of left-behind adolescents. Among them, resilience, self-esteem, positive coping, and negative coping play an important mediating role in the relationship between life events and left-behind adolescents’ aggressive behaviors. Left-behind adolescents with high resilience, self-esteem, and positive coping tendency are less likely to have aggressive behaviors. Therefore, parents, teachers, and other guardians should not only avoid the negative impact of life events on left-behind adolescents but also emphasize the development of their self-awareness and evaluation ability. Their resilience, self-esteem, and positive coping tendency can be improved by strengthening their psychological support and positive guidance, thus reducing or preventing their aggressive behavior and promoting their physical and mental health.

## Data availability statement

The original contributions presented in this study are included in this article/supplementary material, further inquiries can be directed to the corresponding authors.

## Ethics statement

The studies involving human participants were reviewed and approved by the Behavioral Medicine and Nursing Ethics Review Committee, School of Nursing, Central South University. Written informed consent to participate in this study was provided by the participants’ or their legal guardian/next of kin.

## Author contributions

JeZ and YL designed the study, analyzed the data, interpreted the results, and wrote the manuscript. YC and JL interpreted the data, prepared the manuscript, and revised the manuscript. YT and BY conducted the statistical analysis and interpreted the result. SY and CT collected the data and revised the manuscript. JnZ conducted the statistical analysis and provided the consultation in the study design process. FC and TM designed the study, analyzed the data, interpreted the results, and provided the consultation in the study design process. All authors have read and reviewed the final manuscript.

## Footnotes

1
https://www.danielsoper.com/statcalc/calculator.aspx?id=89
